# Deconstructing the Emotion Regulatory Properties of Mindfulness: An Electrophysiological Investigation

**DOI:** 10.3389/fnhum.2016.00451

**Published:** 2016-09-07

**Authors:** Yanli Lin, Megan E. Fisher, Sean M. M. Roberts, Jason S. Moser

**Affiliations:** Department of Psychology, Michigan State UniversityEast Lansing, MI, USA

**Keywords:** mindfulness, emotion, LPP, meditation, ERPs

## Abstract

The present study sought to uncover the emotion regulatory properties of mindfulness by examining its effects—differentiated as a meditative practice, state of mind and dispositional trait—on the late positive potential (LPP), an event-related potentials (ERPs) indexing emotional processing. Results revealed that mindfulness as a meditative practice produced a reduction in the difference between the LPP response to negative high arousing and neutral stimuli across time. In contrast, a state mindfulness induction (i.e., instructions to attend to the stimuli mindfully) failed to modulate the LPP. Dispositional mindfulness, however, was related to modulation of the LPP as a function of meditation practice. Dispositional mindfulness was associated with a reduction of the LPP response to negative high arousal stimuli and the difference between negative high arousal and neutral stimuli in participants who listened to a control audio recording but *not* for those who engaged in the guided meditation practice. Together, these findings provide experimental evidence demonstrating that brief mindfulness meditation, but not deliberate engagement in state mindfulness, produces demonstrable changes in emotional processing indicative of reduced emotional reactivity. Importantly, these effects are akin to those observed in individuals with naturally high dispositional mindfulness, suggesting that the benefits of mindfulness can be cultivated through practice.

## Introduction

Mindfulness, commonly referred to as a form of nonjudgmental present-focused attention (FA; Kabat-Zinn, 1990), has gained worldwide popularity as a distinct method to promote health and well-being. A rapidly growing body of scientific research, has indeed, demonstrated the salutary effects of mindfulness on cognition (Jha et al., 2010; MacLean et al., 2010), physiology (Davidson et al., 2003; Carlson et al., 2007) and psychological well-being (Chiesa and Serretti, 2009; Keng et al., 2011). The proliferation of research documenting the benefits of mindfulness has led to its integration in a variety of efficacious psychotherapeutic interventions (see Baer, 2003; Tapper et al., 2009; Hofmann et al., 2010; Brewer et al., 2011). One explanatory mechanism for the therapeutic benefits of mindfulness involves its effects on emotion regulation (Lutz et al., 2008; for a review see Chambers et al., 2009), a critical self-control ability that is altered in many forms of psychopathology (Kring and Bachorowski, 1999; Aldao et al., 2010). Although much work has uncovered the emotion regulatory benefits of mindfulness, less is known about its associated neural mechanisms.

Uncovering mechanisms of action is complicated by the fact that mindfulness is not a clear monolithic construct. Both Buddhist scholars and contemplative scientists have expounded on the ever-evolving, multifaceted meaning of mindfulness (Bishop et al., 2004; Gethin, 2011; Williams and Kabat-Zinn, 2011; Vago and Silbersweig, 2012). Vago and Silbersweig (2012) cogently summarized the definitional variation within the context of psychological science, referring to mindfulness as a state, trait, meditation practice and a psychological intervention. Such “construct heterogeneity” introduces significant theoretical and design challenges against conducting rigorous experimental studies.

A further complication is that there exists substantial variation in meditative styles and non-meditative behaviors that cultivate mindfulness (Langer, 2014; Lutz et al., 2015). Recent scholarship has distinguished mindfulness-based meditative practices into two broad forms (Lutz et al., 2008): (1) FA meditation, which involves directing and sustaining attention on a selected object; and (2) Open Monitoring (OM) meditation that entails nonreactive, meta-cognitive monitoring of the present moment experience. Although FA and OM meditation involve many overlapping elements, each form is conceptualized to train distinctive cognitive abilities. Specifically, OM meditation may contain more emotion regulatory properties than FA meditation because of its focus on fostering nonreactive awareness as opposed to sustained attention (Lutz et al., 2008, 2014; Perlman et al., 2010; Fox et al., 2016). On the other hand, Langer’s (2014) work exemplifies cultivating mindfulness through non-meditative means, demonstrating that it is both practically feasible and beneficial to engage the world mindfully during everyday activities. These differences among meditative and non-meditative mindfulness modalities underscore the importance for researchers to recognize mindfulness as a form(s) of meditation *and* an experiential state of mind, and to skillfully account for these differences within the context of the research question.

In response to these challenges, the field of contemplative science has called on researchers to move towards an integrative neuroscientific approach that embraces the conceptual nuances of mindfulness (Hölzel et al., 2011; Vago and Silbersweig, 2012; Lutz et al., 2015). Consonant with the spirit of this movement, we sought to develop a more nuanced mechanistic understanding of the emotion regulatory properties of mindfulness by deconstructing it as a meditation exercise, state of mind, and dispositional trait using a neurocognitive approach. Because temporality is a core feature of emotion regulation (Davidson, 2004; Gross and Thompson, 2007; Sheppes and Gross, 2011), a well-suited way to examine the effects of mindfulness on emotion regulation is through the use of event-related potentials (ERPs)—electrophysiological brain signals that reflect event or stimulus-locked neural activity with millisecond temporal precision.

One particularly relevant ERP is the visually evoked late positive potential (LPP), a centro-parietally maximal positive deflection that reaches maximum amplitude 300–800 ms after the onset of emotionally evocative stimuli and lasts for several seconds. The LPP has been shown to index the motivational relevance of visual stimuli such that its amplitude corresponds to the arousal level of affective stimuli—exhibiting more positivity (i.e., larger amplitude) for more emotionally evocative stimuli (Cuthbert et al., 2000; Schupp et al., 2000; Keil et al., 2002; Hajcak et al., 2012). Early time windows (300–1000 ms) are thought to index bottom-up attention allocation (Olofsson et al., 2008), whereas later time windows (>1000 ms) index semantic processing (Foti and Hajcak, 2008; MacNamara et al., 2009). Furthermore, early and late LPP time windows are sensitive to various emotion regulation strategies (Moser et al., 2006, 2009, 2014; Thiruchselvam et al., 2011), and correlate with self-reported changes in emotional intensity during regulation (Hajcak and Nieuwenhuis, 2006).

Recent research by Brown et al. (2013) and Sobolewski et al. (2011) exemplify the advantages of using an electrophysiological approach to study mindfulness. Both groups found an association between mindfulness and reduced emotional responsitivity to unpleasant emotionally evocative stimuli using the LPP. Specifically, Brown et al. (2013) found that higher dispositional mindfulness correspond to smaller early LPP responses elicited by negative arousing pictures, suggesting that trait mindfulness may attenuate early responding to emotionally salient stimuli. In line with these findings, Sobolewski et al. (2011) found a smaller difference between LPPs elicited by negative arousing and neutral stimuli in experienced meditators relative to controls, indicating that mindfulness meditation practice may attenuate emotional reactivity to aversive stimuli. These conclusions are notably consistent with fMRI studies reporting an association between mindfulness and downregulation of emotional brain regions (Creswell et al., 2007; Goldin and Gross, 2010; Modinos et al., 2010; Lutz et al., 2014).

Although these two studies provide an illuminating first look at the temporal dynamics of mindfulness effects on emotional processing, the ability to draw meaningful conclusions is restricted due to several notable limitations. First, both studies were quasi-experimental, examining how preexisting differences in dispositional mindfulness and meditation experience relate to the LPP. An experimental design is needed to draw causal inferences and rule out alternative hypotheses related to selection factors and confounding dispositional variables. Second, both groups directed their analyses to relatively early time windows (Brown et al. (2013): 500–900 ms, Sobolewski et al. (2011): 0–1500 ms), limiting our understanding of the effects of mindfulness during later “meaning-making” stages when the LPP is particularly sensitive to cognitive emotion regulation strategies (Foti and Hajcak, 2008; MacNamara et al., 2009; Moser et al., 2014). Third, neither study accounted for the polylithic nature of mindfulness—separately examining mindfulness as a trait and meditation practice without accounting for the possibility that different forms of mindfulness may have distinct and or interactive effects on emotional processing.

Consequently, the present study sought to address these limitations and fill the gaps in knowledge by adopting an experimental electrophysiological design. Specifically, our investigation had three aims: (1) to provide experimental evidence for the effects of mindfulness on emotional processing of negative and neutral stimuli via the LPP and the negative-neutral difference wave; (2) to examine the effects of mindfulness on early (<1000 ms) and later stages of emotional processing (>1000 ms); and (3) to account for construct heterogeneity by differentially operationalizing mindfulness as a form of OM meditation exercise, state of mind and dispositional trait.

## Materials and Methods

### Participants

Sixty-eight native English speaking, right-handed, female undergraduates participated in the study for course credit. Given that there are sex differences in emotional reactivity to negative stimuli—namely, higher arousal and larger LPP amplitudes relative to men—we recruited only females to minimize the moderating confound of sex (Bradley et al., 2001; Syrjänen and Wiens, 2013). Furthermore, because women are more susceptible to mood and anxiety disorders (Seedat et al., 2009; McLean et al., 2011) and have been shown to regulate emotions differently than men (Gross and John, 2003; McRae et al., 2008), examining the emotion regulatory properties of mindfulness in an all-female sample could yield valuable clinical insights.

Prospective participants were screened for a history of neurological illness and meditation experience. We recruited only novices to reduce confounds associated with meditation experience. Consented participants were then randomly assigned to a group involving a unique combination of audio recording type and viewing instruction (see below for details). Participants randomized to the control group (*n* = 16) listened to the control audio and were instructed to view the pictures naturally. Those in the mindful viewing group (*n* = 14) listened to the control audio, but were instructed to view the pictures mindfully. In contrast, the mindful meditation group (*n* = 17) engaged in a guided audio meditation and was instructed to attend to the picture naturally. Lastly, the mindful meditation and viewing group (*n* = 21) engaged in the guided meditation and was instructed to view pictures mindfully. Eleven participants were excluded from the analyses due to excessive electroencephalogram (EEG) artifacts (rates that resulted in fewer than eight usable trials for at least one image valence condition; see Moran et al., 2013). The remaining 57 participants (control: *n* = 12, mindful viewing: *n* = 10, mindful meditation: *n* = 15, mindful meditation and viewing: *n* = 20) that comprised the final sample ranged in age from 18 to 22 (*M* = 19.18, *SD* = 1.21). All participants were naïve to group assignments throughout the entire duration of the experiment.

### Procedural Overview

The Institutional Review Board at Michigan State University approved the study procedures and all participants provided written, informed consent prior to participation. After consenting, participants were fitted with an electrode cap for EEG recording. Participants were then randomly assigned to engage in a guided audio meditation exercise or listen to a control audio clip (this experimental variable will be referred to as “audio type”). Immediately following the audio induction, participants engaged in a picture viewing task while continuous EEG was recorded. During picture viewing, participants were randomly instructed to attend to the pictures either naturally or mindfully (henceforth referred to as “viewing instruction”). Upon finishing the task, the EEG equipment was removed and participants completed a self-report mindfulness questionnaire and a manipulation check measure, both of which are described in the “Measures” Section below. Five other self-report measures were collected for a separate study.

### Tasks, Materials and Instructions

#### Audio Induction

The OM meditation training was comprised of a 20 min guided meditation exercise (Hickman) led by Steve Hickman from the University of San Diego Center for Mindfulness. The recording, instructed listeners to attend to their present-moment experience in an open, nonjudgmental way, taking notice of any arising feelings, thoughts, or physical sensations. Listeners were instructed to orient to their breath when attention wavered. This guided meditation was selected because of its primary emphasis on attending to various sensory experiences (a defining feature of OM), as opposed to other audio exercises aimed at training sustained attention to the breath (e.g., Larson et al., 2013).

The control audio was an 18-min recording of a TED talk by the linguist Lonsdale (2013). The recording teaches listeners how to quickly acquire second language fluency. The clip was selected to match the duration, didactic nature, gender, and speaking style of the guided meditation clip.

#### Viewing Instructions

Participants were instructed to view the pictures mindfully or naturally based on group assignments. Mindful viewing instructions involved directing participants to be aware of the sensations, thoughts, and emotions that arose during picture viewing. Participants were told to attend to these internal experiences impartially, without judgment or self-elaboration—observing without trying to change, suppress, or avoid. Natural viewing involved telling the participant to simply attend to the pictures naturally and respond as they normally would, without specifying the definition of “natural”.

#### Picture Viewing Task

Stimuli included 90 pictures taken from the International Affective Picture System (IAPS; Lang et al., 2008). Consistent with Brown et al. (2013), the images were selected and organized into three equal groups varying on valence and arousal ratings: 30 negative (*M* = 2.22), high arousing (*M* = 6.63) pictures; 30 negative (*M* = 3.41), low arousing (*M* = 4.13) pictures; and 30 neutral (*M* = 4.76), low arousing (*M* = 3.20) pictures^1^. Negative images were split by arousal to detect potential interactive effects between mindfulness and arousal on emotion processing. However, we expected to detect stronger effects on high arousal images because the LPP has been shown to be more sensitive to more arousing stimuli (Cuthbert et al., 2000; Schupp et al., 2000). The stimuli were presented on a Pentium R Dual Core computer using E-Prime software (Psychology Software Tools, Inc., Sharpsburg, PA, USA) to control the timing and duration of the images. Each image was displayed full screen in color on a 19″ flat-screen LCD monitor approximately 41″ from the participant.

On each trial, a white fixation cross (+) was presented at the center of the screen for 500 ms. A randomly selected image was then displayed on the entire screen for 5000 ms. The inter-trial interval between image offset and fixation onset varied randomly between 2000–4000 ms. Presentation of the 90 images were divided into three blocks of 30 trials, with each block containing 10 negative low arousal, 10 negative high arousal and 10 neutral images.

### Measures

#### Mindfulness

The 39-item Five-Factor Mindfulness Questionnaire (FFMQ; Baer et al., 2006), a psychometrically validated scale that distinguishes mindfulness into five unique facets, was used to assess trait mindfulness. The five factors involve: (a) observing, defined as noticing or attending to internal and external experiences; (b) describing, defined as verbal labeling of internal experiences; (c) acting with awareness, defined as attending to present moment experience; (d) nonjudging, defined as adopting a nonevaluative perspective toward thoughts and feelings; and (e) nonreactivity, defined as allowing inner experiences (e.g., thoughts and feelings) to come and go without attachment or elaboration. Participants respond to each item using a 5-point Likert scale ranging from 1 (*never or very rarely true*) to 5 (*very often or always true*). Analyses focused on the Acting with Awareness subscale (henceforth referred to as FFMQ awareness) due to its specificity as a dispositional measure of mindful awareness rather than technical abilities cultivated during mindfulness meditation (Brown and Cordon, 2009; Goodman et al., 2015). Consequently, FFMQ awareness (*α* = 0.84) was an applicable measure for all participants, including those randomized to the non-meditative groups. Importantly, the strong correlation (*r* = 0.89; Baer et al., 2006) between FFMQ awareness and the Mindful Attention Awareness Scale (MAAS; Brown and Ryan, 2003)—another trait measure of mindfulness—further supports the use of FFMQ awareness as a dispositional measure.

#### Manipulation Check Questionnaire

To assess for differences in participant engagement and reception to the audio induction and picture viewing instructions, participants completed a post-task manipulation check questionnaire. Participants rated the extent to which they found the audio induction engaging, interesting, and arousing (1 = *not at all*, 7 = *very*). Participants were also asked to indicate their level of comprehension (1 = *did not understand*, 7 = *completely understand*), emotional reaction (1 = *very negative*, 4 = *neutra*l, 7 = *very positive*) and whether they learned anything (1 = *very little*, 7 = *very much*). Likewise, participants rated the extent to which they understood the viewing instructions (1 = *no understanding*, 7 = *full understanding*) and were able to follow them (1 = *not effectively*, 7 = *very effectively*). Participants also rated the overall interest in the task (1 = *not interesting*, 7 = *very interesting*), as well as their reactivity (1 = *no reaction*, 7 = *high reaction*) and attention (1 = *not attentive*, 7 = *very attentive*) to the pictures. Lastly, depending on audio type, participants were asked how likely they were to start meditation or learn a second language (1 = *not likely*, 7 = *very likely*).

#### Electrophysiological Recording and Data Reduction

The EEG was recorded using active Ag/AgCl electrodes (BioSemi ActiveTwo) placed at the left and right mastoids and 64 scalp sites according to the modified 10–20 system. The electrooculogram (EOG) was recorded from electrodes placed at the outer canthi of each eye, and above and below the left eye. The EEG and EOG signals were digitized at 1024 Hz.

Off-line, the EEG signals were re-referenced to the average of the left and right mastoids. Ocular artifacts were corrected using the algorithm developed by Gratton et al. (1983). All signals were low-pass filtered at 20 Hz and high-pass filtered at 0.01 Hz. EEG epochs of 5200 ms (200 ms baseline) were extracted from the continuous data file for analysis. Trials were automatically excluded based on the following criteria: a voltage step of more than 50 μV between sample points, a voltage difference of more than 400 μV within 200 ms intervals, voltage exceeding ±200 μV, and a maximum voltage difference of less than 0.50 within 1000 ms intervals.

As in previous work (Moser et al., 2014), we partitioned the LPP based on early and late time windows in order to examine the effects of the experimental manipulations on early automatic attention processing and later semantic processing, respectively. We defined the electrophysiological activity during the early window as the *early maximal LPP*, quantified as the average voltage in the 600–1000 ms time window during which the LPP was maximally positive. The late window response, defined as the *late sustained LPP*, was quantified as the average voltage within successive 1000 ms time windows ranging from 1000 to 5000 ms post stimulus onset. This division of the LPP was further supported by visual inspection of the ERP waveform across electrode sites showing that differences in amplitude by stimulus type (neutral, negative low arousal, negative high arousal) and experimental condition were most salient after approximately 1 s following stimulus onset and persisted until the end of the viewing period. Consistent with previous research showing that the LPP is largest at central and parietal midline sites (e.g., Cuthbert et al., 2000; Schupp et al., 2000), the LPP was calculated at electrode site CPz, where amplitude was maximal in the current study. Notably, Brown et al. (2013) also conducted their analyses using signal values extracted from CPz. By maintaining methodological consistency, we aimed to maximize cross-study generalizability.

## Results

### Manipulation Check

Submitting participant responses to the audio recording to independent-samples *t*-tests with audio type (meditation, control) as a group variable revealed differences in interest (*t*_(1,55)_ = 3.54, *p* < 0.01) and learning (*t*_(1,55)_ = 3.65, *p* < 0.01), such that participants who listened to the control audio rated the induction as more interesting (*M* = 4.05, *SD* = 1.59) and endorsed learning more (*M* = 4.82, *SD* = 1.37) relative to participants who engaged in the guided meditation (interest: *M* = 2.63, *SD* = 1.40, learning: *M* = 3.43, *SD* = 1.42). Importantly, however, there were no differences in task engagement, affective reactivity, arousal, or understanding (*t*s < 1.44, *p*s > 0.15), suggesting that participants approached and responded to the audio inductions similarly on these other dimensions.

Participant responses to the picture viewing task were likewise submitted to independent-samples *t*-tests with viewing instruction (natural, mindful) as a group factor. Differences emerged in participant understanding (*t*_(1,55)_ = 2.75, *p* = 0.01) and the ability to follow instructions (*t*_(1,55)_ = 2.54, *p* = 0.01), such that natural viewing instructions were easier to understand (*M* = 6.74, *SD* = 0.53) and follow (*M* = 6.44, *SD* = 0.89) compared to mindful viewing instructions (understanding: *M* = 6.07, *SD* = 1.16, follow instruction: *M* = 5.70, *SD* = 1.26). Despite the statistically significant difference (possibly driven by a ceiling effect for natural viewing), mean ratings were high for both viewing conditions (*t*s > 9.54, *p*s < 0.01 using a one-sample *t*-test against the midpoint value of 3.5), suggesting adequate task compliance. Importantly, there were no differences in interest, reactivity, or attention (*t*s < 1.3 *p*s > 0.20) to the pictures as a function of viewing instruction.

To examine group differences in dispositional mindfulness, FFMQ scores were submitted to a one-way ANOVA with group assignment (i.e., audio type, viewing instruction) as a between subject factor. No group differences emerged for any of the five factors or overall mindfulness score (*F*s < 1.8, *p*s > 0.16). Together, these results suggest that differences in participants’ understanding of and adherence to the experimental conditions were minimal and any observed differences in the LPP are unlikely attributable to experimental or trait related confounds.

### Effects of Experimental Manipulations on LPP Amplitudes by Stimulus Type

**Figure 1:**
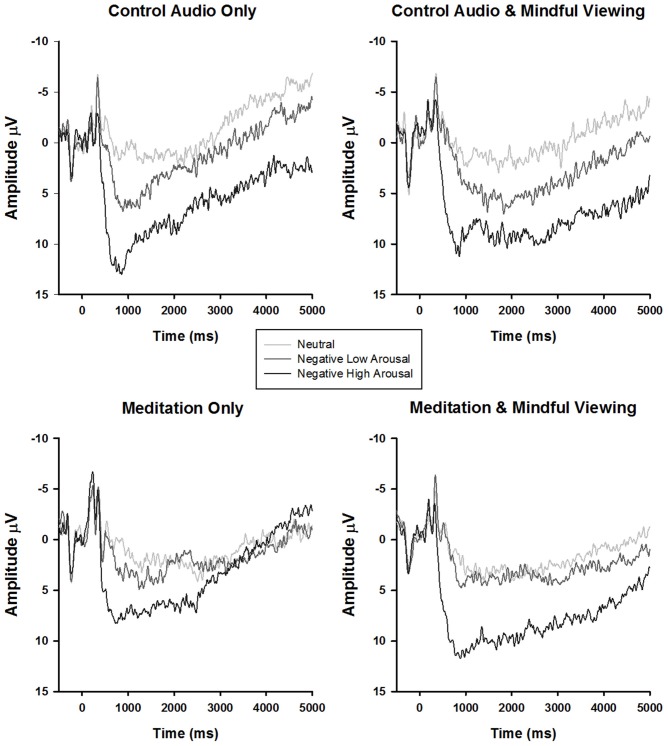
**Stimulus synchronized grand-average event-related potential (ERP) waveforms elicited by stimulus type and separated by experimental condition at electrode site CPz**.

ERP waveforms and amplitude across the four experimental conditions are presented in Figure 1 and Tables 1, 2, respectively. To analyze the effects of audio type and viewing instructions, the early maximal LPP was submitted to a 3 stimulus type (negative high arousal, negative low arousal, neutral) one-factor repeated-measures ANOVA (rANOVA) with audio type (meditation, control) and viewing instruction (mindful, natural) as between subject factors. Likewise, the late sustained LPP was submitted to a 3 stimulus type (negative high arousal, negative low arousal, neutral) × 4 time (1000–5000 ms) rANOVA with audio type and viewing instruction as between subject factors. Main and interactive effects involving time and stimulus type were explored using within-subject contrasts. Greenhouse-Geisser corrections were applied to *p*-values associated with multiple *df* repeated measures comparisons when appropriate.

**Table 1:** **Summary of early maximal late positive potential (LPP) amplitudes at the CPz electrode site by experimental condition**.

Experimental condition	Control		Mindful viewing		Meditation only		Meditation and mindful viewing	
Amplitude (μV)	*M*	*SD*	*M*	*SD*	*M*	*SD*	*M*	*SD*
Neutral (600–1000 ms)	0.71	5.76	0.61	5.08	0.43	7.25	1.67	4.49
Negative low arousal (600–1000 ms)	5.17	5.56	1.95	5.55	2.50	8.70	3.03	4.87
Negative high arousal (600–1000 ms)	12.02	7.09	9.47	5.53	7.61	8.64	10.76	7.27
Negative high neutral difference (600–1000 ms)	11.31	6.40	8.86	3.00	7.18	5.80	9.09	5.88

**Table 2:** **Summary of late sustained LPP at the CPz electrode site by experimental condition**.

Experimental condition	Control		Mindful viewing		Meditation only		Meditation and mindful viewing	
Amplitude (μV)	*M*	*SD*	*M*	*SD*	*M*	*SD*	*M*	*SD*
Neutral (1000–2000 ms)	1.12	6.00	1.50	4.72	2.08	6.60	3.12	4.59
Negative low arousal (1000–2000 ms)	4.40	4.02	5.30	4.21	3.51	7.87	3.83	3.82
Negative high arousal (1000–2000 ms)	8.88	6.01	8.81	4.52	6.76	8.68	10.07	6.43
Neutral (2000–3000 ms)	0.55	5.72	1.16	4.62	2.61	5.01	3.06	6.63
Negative low arousal (2000–3000 ms)	1.90	5.16	4.92	5.80	2.16	8.94	3.56	4.95
Negative high arousal (2000–3000 ms)	6.32	8.61	9.23	6.20	5.42	11.25	8.97	7.11
Neutral (3000–4000 ms)	−3.28	6.28	−0.76	5.55	0.77	6.64	1.69	6.92
Negative low arousal (3000–4000 ms)	−0.46	6.86	2.74	7.92	1.90	8.87	3.08	6.68
Negative high arousal (3000–4000 ms)	4.40	8.80	7.32	6.83	1.79	11.74	7.68	7.40
Neutral (4000–5000 ms)	−5.36	6.90	−2.80	7.52	−0.32	7.90	0.21	6.36
Negative low arousal (4000–5000 ms)	−2.98	8.90	0.40	7.76	−0.29	8.90	1.93	7.61
Negative high arousal (4000–5000 ms)	2.43	9.22	5.81	5.46	−1.85	11.30	5.20	7.66
Negative high neutral difference (1000–2000 ms)	7.76	7.73	7.32	3.82	4.68	9.61	6.96	6.24
Negative high neutral difference (2000–3000 ms)	5.77	9.15	8.07	4.17	2.81	10.44	5.91	7.79
Negative high neutral difference (3000–4000 ms)	7.68	8.45	8.09	4.96	1.01	8.93	5.98	7.61
Negative high neutral difference (4000–5000 ms)	7.80	11.16	8.61	7.15	−1.53	10.59	4.99	8.41

#### Early Maximal LPP

Consistent with previous research, a main effect of stimulus type emerged (*F*_(1.87,98.98)_ = 89.92, *p* < 0.01, $\eta_{p}^{2}\;$ = 0.63). Follow-up tests of within-subjects contrasts revealed a linear trend such that the LPP exhibited more positivity for negative arousing stimuli, than negative low arousing stimuli, and least positivity for neutral stimuli (*F*_(1,53) lin_ = 141.47, *p* < 0.01, $\eta_{p}^{2}\;$ = 0.73). No significant interactions associated with viewing instruction or audio type emerged (*F*s < 1.19, *p*s > 0.31).

#### Late Sustained LPP

For the late sustained LPP, main effects of stimulus type (*F*_(1.95,103.08)_ = 15.45, *p* < 0.01, $\eta_{p}^{2}\;$ = 0.23) and time (*F*_(1.54,81.53)_ = 36.72, *p* < 0.01, $\eta_{p}^{2}\;$ = 0.41) emerged, such that the LPP exhibited more positivity for negative arousing stimuli (*F*_(1,53)lin_ = 31.51, *p* < 0.01, $\eta_{p}^{2}\;$ = 0.37) but reduced positivity over time irrespective of stimulus type (*F*_(1,53)lin_ = 46.35, *p* < 0.01, $\eta_{p}^{2}\;$ = 0.47). Critically, these main effects were qualified by a time × stimulus type × audio type interaction (*F*_(3.05,161.47)_ = 2.83, *p* = 0.04, $\eta_{p}^{2}\;$ = 0.05). No other significant interactions emerged (*F*s < 1.9, *p*s > 0.16).

**Figure 2:**
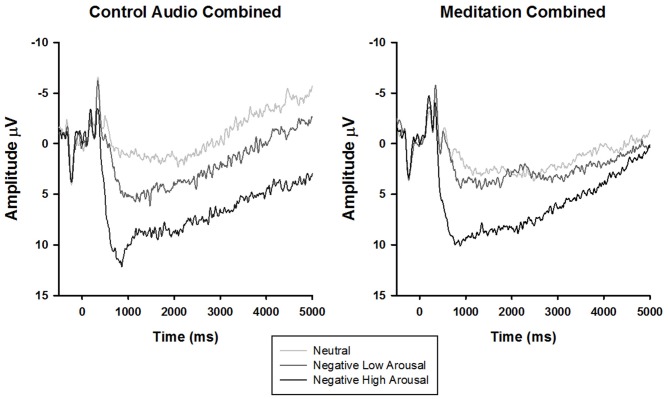
**Stimulus synchronized grand-average ERP waveforms elicited by stimulus type and separated by audio type at electrode site CPz**.

To parse the three-way interaction, the data were split by audio type (meditation, control) and submitted to separate 3 stimulus type × 4 time rANOVAs. In both groups, there was a main effect of time (control: *F*_(1.51,31.77)_ = 25.34, *p* < 0.01, $\eta_{p}^{2}\;$ = 0.55, meditation: *F*_(1.48,50.24)_ = 14.91, *p* < 0.01, $\eta_{p}^{2}\;$ = 0.31) and stimulus type (control: *F*_(1.87,39.34)_ = 11.91, *p* < 0.01, $\eta_{p}^{2}\;$ = 0.36, meditation: *F*_(1.95,66.16)_ = 5.49, *p* < 0.01, $\eta_{p}^{2}\;$ = 0.14). Interestingly, however, a significant time × stimulus type interaction emerged only for the meditation (*F*_(2.80,95.17)_ = 3.73, *p* = 0.02, $\eta_{p}^{2}\;$ = 0.10), but *not* control group (*F*_(3.24,68.11)_ = 0.51, *p* = 0.80, $\eta_{p}^{2}\;$ = 0.02). Follow-up tests of within-subjects contrasts demonstrated that the time × stimulus type interaction in the meditation group exhibited a linear trend (*F*_(1,34)lin×lin_ = 6.05, *p* = 0.02, $\eta_{p}^{2}\;$ = 0.15), such that the difference in LPP amplitude by stimulus type diminished linearly over time (i.e., larger amplitudes elicited by more negative arousing stimuli decreased across time to neutral stimuli amplitude levels; see Figure 2).

### Relationship Between LPP and Dispositional Mindfulness

To examine the role of dispositional mindfulness on the early maximal and late sustained LPP, the electrophysiological data were submitted to separate ANOVAs: a single-factor 3 stimulus type and a 3 stimulus type × 4 time rANOVA, respectively. Each rANOVA included view instruction and audio type as between subject factors and FFMQ awareness scores as a covariate. Follow-up correlational analyses involving the LPP by stimulus type and the negative-neutral difference were conducted to parse emerging interactions.

#### Early Maximal LPP

The rANOVA revealed a significant stimulus type × audio type × FFMQ awareness interaction (*F*_(1.85,90.44)_ = 3.15, *p* = 0.05, $\eta_{p}^{2}\;$ = 0.06). We parsed this three-way interaction by splitting the data by audio type. In the control group, a main effect of stimulus type (*F*_(1.81,36.25)_ = 8.43, *p* < 0.01, $\eta_{p}^{2}\;$ = 0.30) was qualified by a stimulus type × FFMQ awareness interaction (*F*_(1.81,36.25)_ = 3.23, *p* = 0.06, $\eta_{p}^{2}\;$ = 0.14). In contrast, there were no main or interactive effects in the meditation group (*F*s < 1.16, *p*s > 0.31, $\eta_{p}^{2}\;$ < 0.03).

**Figure 3:**
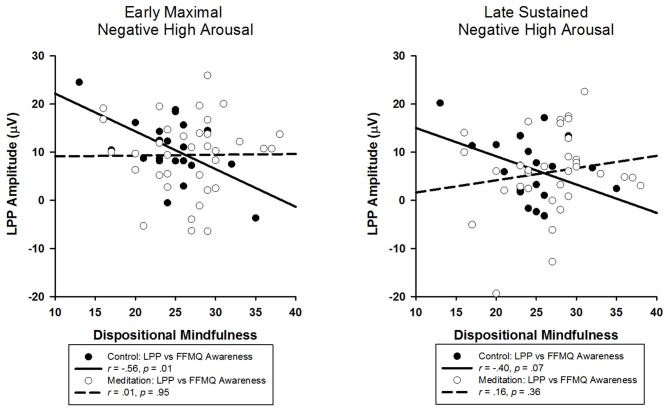
**Scatter-plots depicting LPP amplitudes elicited by negative high arousal images and Five-Factor Mindfulness Questionnaire (FFMQ) awareness as a function of audio type and separated by time window**.

The correlational analyses revealed that a smaller LPP elicited by negative high arousing stimuli was associated with higher scores on the FFMQ awareness scale in the control (*r* = −0.56, *p* = 0.01), but *not* meditation group (*r* = 0.01, *p* = 0.95; see Figure 3). Notably, the magnitude of the control group correlation was nearly identical to that reported by Brown et al. (2013). With the exception of an unpredicted marginal correlation between FFMQ awareness and neutral LPPs in the control group (*r* = −0.41, *p* = 0.06), FFMQ awareness was not significantly correlated with any other LPP in either group (*r*s < |0.27|, *p*s > 0.22).

#### Late Sustained LPP

The rANOVA revealed a time × stimulus type × audio type interaction (*F*_(3.07,150.36)_ = 2.87, *p* = 0.04, $\eta_{p}^{2}\;$ = 0.06), mimicking the primary finding (above) that audio type modulated the late sustained LPP even after controlling for mindful awareness. Furthermore, a stimulus type × audio type × FFMQ awareness interaction (*F*_(1.98,97.14)_ = 6.15, *p* < 0.01, $\eta_{p}^{2}\;$ = 0.11) also emerged. No other significant main or interactive effects emerged (*F*s < 2.20, *p*s > 0.09).

We parsed the three-way stimulus type × audio type × FFMQ awareness interaction by aggregating the data across time and splitting by audio type. In the control group, a main effect of stimulus type (*F*_(1.92,38.40)_ = 10.63, *p* < 0.01, $\eta_{p}^{2}\;$ = 0.35) was qualified by a stimulus type × FFMQ awareness interaction (*F*_(1.92,38.40)_ = 7.55, *p* < 0.01, $\eta_{p}^{2}\;$ = 0.27). In contrast, there were no main or interactive effects in the meditation group (*F*s < 1.16, *p*s > 0.31, $\eta_{p}^{2}\;$ < 0.03).

**Figure 4:**
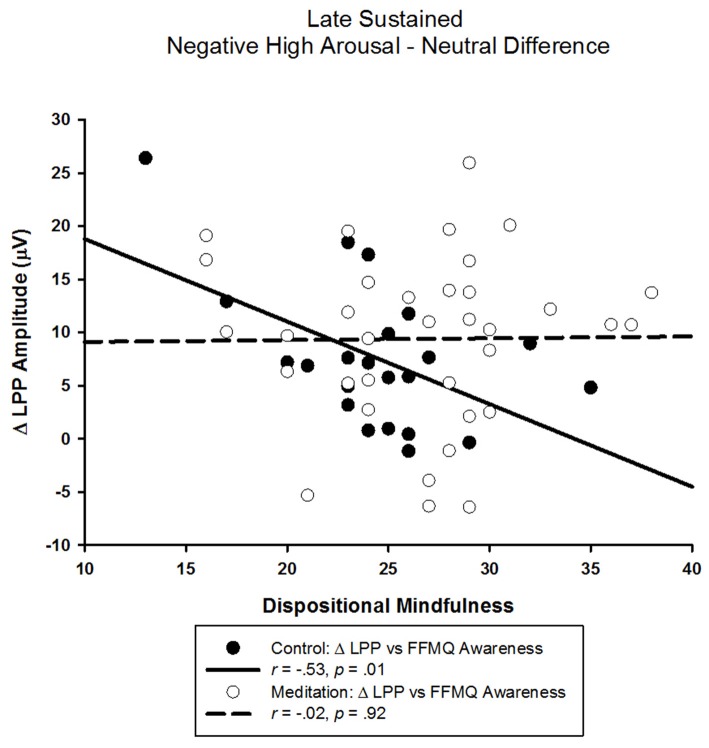
**Scatter-plot depicting the LPP amplitude difference between negative high arousal and neutral stimuli and FFMQ awareness as a function of audio type**.

The correlational analyses in the control group showed that a smaller LPP elicited by negative high arousing pictures was associated with higher FFMQ awareness scores (*r* = −0.40, *p* = 0.07). Similarly, a smaller difference between LPPs elicited by negative high arousing and neutral pictures was associated with higher FFMQ awareness scores (*r* = −0.53, *p* = 0.01; see Figure 4). Neither the LPP elicited by neutral pictures nor the difference between the LPP elicited by negative low arousing pictures and neutral pictures were related to FFMQ awareness (*r*s < 0.26, *p*s > 0.24). Unexpectedly, we also found that a larger LPP elicited by negative low arousing pictures was associated with higher FFMQ awareness (*r* = 0.46, *p* = 0.03). As indicated by the lack of ANOVA effects above, FFMQ awareness was not correlated with any LPP in the meditation group (*r*s < |0.26|, *p*s > 0.14).

## Discussion

Although a growing body of research has linked many of the salutary benefits of mindfulness to its emotion regulatory properties (Chambers et al., 2009), the neural mechanisms involved in its effects on emotion processing remain unclear. Consequently, the present research sought to fill this gap in knowledge by examining how mindfulness influences neurophysiological markers of emotional responding over time. Moreover, the study was designed specifically to address common challenges and limitations involved in mindfulness research. First, we differentially operationalized mindfulness as a meditation exercise, state of mind, and dispositional trait in order to parse the nuances associated with construct heterogeneity (Hölzel et al., 2011; Vago and Silbersweig, 2012; Lutz et al., 2015). Second, we adopted an experimental design to facilitate the drawing of causal inferences and to corroborate previous ERP findings associating mindfulness with lower LPP responses to aversive stimuli (Sobolewski et al., 2011; Brown et al., 2013). Lastly, our analyses extended to later time windows in order to derive a more comprehensive understanding of the role of mindfulness during later stages of emotional processing.

Examining the effects of the experimental manipulations during the early time window revealed no modulation of the early maximal LPP, suggesting that neither the OM meditation exercise nor mindful viewing instruction influenced early emotional stimulus processing. Given that the early maximal LPP is theorized to index bottom-up allocation of attention (Olofsson et al., 2008; Hajcak et al., 2012), the absence of its modulation may indicate that a brief one-time mindfulness intervention is insufficient to significantly alter the “automaticity” of early affective processing. Given that previous research has demonstrated that intensive meditation can modulate “bottom-up” attentional processing (Slagter et al., 2007), longitudinal designs involving longer periods of meditation (e.g., Carlson et al., 2007) may be fruitful towards testing whether the early LPP response can change as a function of longer-term mindfulness practice.

However, correlational analysis showed that the early LPP elicited by negative high arousal stimuli was, in fact, smaller for more trait mindful individuals, replicating Brown et al. (2013) finding. Importantly, this near exact replication was only observed for participants assigned to the control audio, but *not* for those who engaged in the guided meditation, suggesting that although the guided meditation did not modulate the early LPP, it was robust enough to modulate the preexisting relationship between dispositional mindfulness and early emotional processing.

Extending the analyses to later time windows revealed that the sustained LPP was modulated by audio type but not viewing instruction. Specifically, the difference in LPP amplitude between the negative and neutral stimuli attenuated linearly over time for the guided meditation but not the control audio group. This finding is particularly intriguing given that previous studies have shown that mass repetition of picture viewing does not modulate the amplitude difference between negative and neutral stimuli (Codispoti et al., 2006; Ferrari et al., 2011). Consequently, it follows that the guided meditation influenced emotional processing in ways that differ markedly from habituation—perhaps through “dampening” underlying systems that allocate enhanced motivational significance to aversive visual representations (Lang and Bradley, 2010). Interestingly, this effect emerged despite self-reported differences in interest and learning—that is, participants who listened to the TED talk rated it as more interesting and endorsed learning more relative to the participants who engaged in the guided meditation. This indicates that the emotional regulatory properties of meditation may not be contingent upon motivational interest or perceived benefit, helping rule out alternative hypotheses related to placebo effects. Clinically, larger differences in the LPP response between negative and neutral stimuli have been linked to a number of psychological disorders (Kolassa et al., 2005; Shackman et al., 2007; Franken et al., 2008). In light of this, the current findings suggest that mindfulness meditation may buffer against the development of psychopathology by reducing the affective salience of negative events.

As observed in the early LPP time window, control audio, but not meditation, participants with higher dispositional mindfulness also exhibited smaller late window LPPs elicited by negative high arousal images. That the relationship between dispositional mindfulness and the LPP elicited by high arousing, negative images was only observed in the control audio condition across both early and late time windows suggests the effect of the guided meditation was robust enough to dampen the LPP irrespective of dispositional mindfulness. Additionally, higher dispositional mindfulness was related to smaller differences in amplitude between negative high arousal and neutral stimuli in the control but not the meditation group. Critically, this relationship between dispositional mindfulness and the LPP difference observed in the control group parallels the experimental effect of the guided meditation and suggests that the *guided meditation modulated emotional processing in ways similar to that of naturally mindful individuals*. Together, these findings converge to provide compelling experimental evidence in support of the notion that OM meditation training attenuates emotional reactivity to aversive events (Lutz et al., 2008; Goldin and Gross, 2010) irrespective of individual differences in dispositional mindfulness prior to training.

To our knowledge, these results are the first to experimentally contrast the role of mindfulness as a meditative exercise from a state of mind—affirming the importance of the recent calls to consider construct heterogeneity (Hölzel et al., 2011; Vago and Silbersweig, 2012; Lutz et al., 2015). The LPP was not modulated by viewing instruction across both early and late time windows, indicating that deliberate engagement in state mindfulness did not influence emotional processing in novices. Contrary to the intuitive hypothesis that meditation exercise enhances the emotion regulatory properties of state mindfulness, our results do not support an additive effect. The most parsimonious explanation is that participants failed to understand the mindful viewing instructions and were unsuccessful in engaging in state mindfulness. However, high mean scores on the manipulation check items in conjunction with Lutz et al.’s (2014) successful implementation of similar instructions to elicit attenuated emotion processing is inconsistent with this hypothesis. Alternatively, a related but more nuanced interpretation is that the novices struggle being mindful in demanding situations (e.g., emotional picture viewing task). In other words, the emotion regulatory properties of being mindful may need to be cultivated in a less demanding, minimally distracting environment (e.g., sitting meditation) prior to becoming salutary in “real” time. This is consistent with Lutz et al.’s (2014) speculation that the attenuating effect of state mindfulness on emotional reactivity may have been stronger in their participants with previous meditation experience. This notion is further supported from Taylor et al.’s (2011) finding that self-perceived emotional attenuation during state mindfulness is associated with distinct neural mechanisms as a function of meditative experience. Future research may test this hypothesis by investigating the long-term interaction between meditation and state mindfulness using a longitudinal design.

### Limitations and Future Research

For the purpose of minimizing sex-related confounds on the experimental effects, this study included an all-female sample. Consequently, further research is needed to determine whether the present results generalize to men. Although most of the results were consistent with the purpose of our design and prior research, we found an unexpected *positive* correlation between the late sustained LPP elicited from negative low arousal stimuli and dispositional mindfulness in the control condition. The contrasting directionality in the relationship between dispositional mindfulness and negative high arousal stimuli relative to negative low arousal stimuli introduces the possibility that mindfulness may differentially influence affective processing of negative stimuli as a function of affective intensity—challenging the conventional notion that mindfulness engenders global attenuation of affective processing. Such an interpretation, however, is highly speculative and further investigation is warranted.

Another related observation involves the possible enhancement of the LPP response to neutral and negative low arousal stimuli in the meditation group (see Figure 2). Rather than solely reducing the LPP response to negative high arousal stimuli, the guided OM meditation appears to elicit more sustained positivity to the neutral and negative low arousal stimuli (particularly after 2500 ms), possibly indicating more sustained attentional processing. This visually convergent pattern in the LPP responses is congruent with the construct of equanimity as recently explicated by Desbordes et al. (2015). Equanimity is defined as an even-minded disposition toward all experiences, regardless of origin or affective valence. Specifically, the authors posit a unique role of attention in differentiating meditative equanimity from other forms of emotion regulation, theorizing that equanimity involves an impartial distribution of attention to all stimuli. Although this interpretation is intriguing, our study was not designed to test this hypothesis and the results reported here do not offer statistically meaningful support. However, we hope that our speculations encourage future research on equanimity and the neural underpinnings of mindfulness as a potentially unique form of emotion regulation.

In terms of task parameters, a potential limitation is that the guided meditation was 2 min longer than the control audio. However, we exercised great precaution to match the audio clips on other substantive dimensions such as didactic style, topic neutrality, and speaker gender, while attempting to match the audio length as closely as possible. Moreover, the likelihood that difference in audio length played a major confounding role in the study seems low given that there were no differences in participant ratings of engagement, affective reactivity, arousal and understanding between the two audio conditions. Lastly, given the challenges of recruiting and retaining participants for a four-group design, our study was somewhat limited by small sample sizes in some of the groups (e.g., *n* = 10 in the control group). Future larger-scale studies involving tighter control conditions may therefore be needed to parse out potentially smaller, more nuanced effects.

## Conclusion

The present study sought to examine the neural dynamics of mindfulness, differentiated as a meditative exercise, state of mind and dispositional trait. Our main finding was that a brief guided OM meditation exercise produced a significant reduction in LPP response to negative stimuli over time, supporting the notion that OM mindfulness meditation attenuates emotional reactivity to aversive events (Lutz et al., 2008; Goldin and Gross, 2010). Our interpretations were further bolstered from finding that, consistent with the experimental effect of the guided meditation, higher dispositional mindfulness was also associated with reduced LPP responses to negative high arousal stimuli in the control but not the meditation condition. Together, our findings demonstrate that a short guided meditation produces meaningful changes in emotional processing in ways similar to that of naturally mindful individuals. In contrast, instructions to attend to the stimuli mindfully failed to modulate the LPP, indicating that deliberate engagement in state mindfulness may not be an effective form of emotion regulation in meditation novices. Consequently, the present study not only adds to a rapidly growing literature touting the benefits of being mindful, but reveals that these salutary benefits can be acquired and cultivated through practice regardless of individual differences in dispositional mindfulness.

## Author Contributions

YL designed the study, collected data, performed data analysis, interpreted the data and wrote the manuscript. MEF assisted in study design, data collection, data interpretation and manuscript evaluation. SMMR assisted in data collection, data interpretation and manuscript evaluation. JSM supervised the development of the study, guided data interpretation and helped edit the manuscript.

## Funding

This research was supported by the Mind and Life Institute, Francisco J. Varela Research Award 2014-Varela-Lin.

## Conflict of Interest Statement

The authors declare that the research was conducted in the absence of any commercial or financial relationships that could be construed as a potential conflict of interest.

## Footnotes

^1^In line with other studies dividing negative stimuli by arousal (see Garavan et al., 2001; Smith et al., 2011; Dolcos et al., 2014), negative high arousing stimuli tended to be more negative than negative low arousing stimuli. Consequently, we encourage readers to interpret the difference between these conditions as affective intensity rather than strictly arousal.
